# Genetic Redundancies Enhance Information Transfer in Noisy Regulatory Circuits

**DOI:** 10.1371/journal.pcbi.1005156

**Published:** 2016-10-14

**Authors:** Guillermo Rodrigo, Juan F. Poyatos

**Affiliations:** 1 Instituto de Biología Molecular y Celular de Plantas, CSIC–UPV, Valencia, Spain; 2 Logic of Genomic Systems Laboratory, CNB–CSIC, Madrid, Spain; Rutgers University, UNITED STATES

## Abstract

Cellular decision making is based on regulatory circuits that associate signal thresholds to specific physiological actions. This transmission of information is subjected to molecular noise what can decrease its fidelity. Here, we show instead how such intrinsic noise enhances information transfer in the presence of multiple circuit copies. The result is due to the contribution of noise to the generation of autonomous responses by each copy, which are altogether associated with a common decision. Moreover, factors that correlate the responses of the redundant units (extrinsic noise or regulatory cross-talk) contribute to reduce fidelity, while those that further uncouple them (heterogeneity within the copies) can lead to stronger information gain. Overall, our study emphasizes how the interplay of signal thresholding, redundancy, and noise influences the accuracy of cellular decision making. Understanding this interplay provides a basis to explain collective cell signaling mechanisms, and to engineer robust decisions with noisy genetic circuits.

## Author Summary

There is increasing evidence that the presence of molecular noise greatly influences function in biological systems. This could imply, for instance, that genetic circuits adopt particular architectures in order to reduce noise. On the other hand, noise can be beneficial. Here, we show that this could be the case for the functioning of analog to digital genetic devices, which are commonly found in cellular decision making situations. We use the framework of information theory to illustrate first how noise can enhance information transfer in these devices. In those regimes in which noise is detrimental, we discuss how genetic redundancies allow information to be maximized, and how this effect depends on the specifics of the devices, and the interdependence among them. These results provide overall an additional rationale for genetic redundancies in genomic systems.

## Introduction

The biochemistry of cells determines the operation of biological circuits. This biochemistry is inevitably noisy [1–3] what immediately suggests a limitation to the reliable function of these circuits, and thus many early studies examined how the problem of achieving correct operation could nevertheless be solved. Mechanisms such as kinetic proofreading [4], or integral feedback control [5] emerged then as some fundamental solutions. One might ask, on the other hand, to what extent noise could indirectly represent an advantage. An example is found when cell populations, in which noise leads to phenotypic variability, display heterogeneity in stress responses that represent a crucial element for survival [6].

In a more direct situation, noise can turn into a necessary ingredient to facilitate new classes of behaviors not achievable otherwise [7–11]. These valuable behaviors are typically related to cellular decisions, which essentially involve changes in the expression phenotype. Specific biological circuits were therefore shown to employ noise to induce the expression of transient phenotypes [8], or to switch among distinct stable states [9]. That many of these probabilistic dynamics relate to systems whose actions are susceptible to limiting signal values [12] emphasizes the connection between noise, cellular decisions, and threshold response circuits.

The beneficial aspect of noise also forces us to revisit some of the original arguments on the relationship between stochasticity and the structure of biological systems [13, 14]. In particular, the existence of genetic redundancies was frequently interpreted as a mean to enhance reliability of operation (i.e., noise as a disruptive element). This role appeared in consequence as a plausible rationale for the evolutionary maintenance of several copies of a gene or circuit [15]. Instead, we focus here on redundancy as a genetic architecture that, when coupled to the effect of noise in threshold response circuits, enables unique information-processing functions.

**Fig 1:**
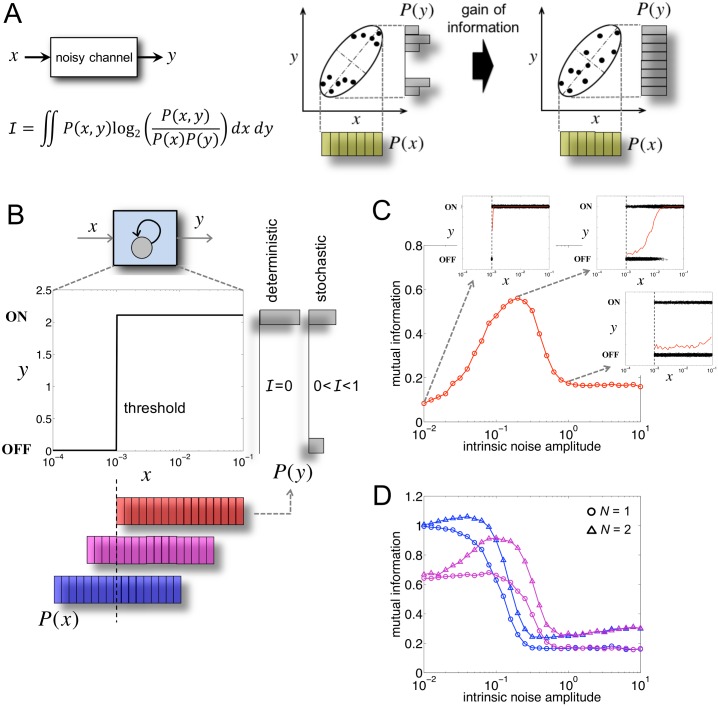
Intrinsic noise can increase or decrease information transfer in threshold genetic systems. (A) Any transmission of signals (*x*) that could lead to an error in the output (*y*) can be framed as a noisy information channel. The mutual information I (MI) quantifies the dependence between input and output distributions, *P*(*x*) and *P*(*y*), respectively, and can discriminate some associations not detected by the correlation coefficient. For instance, the cartoon illustrates two cases with the same correlation (represented by the eccentricity of the ellipses) but different MI. (B) The channel can describe a gene autoactivating its own expression (*y*) in a bistable OFF/ON manner, i.e., a simple example of threshold regulatory circuit. Information transfer depends on the relationship between *x* and the threshold value of activation (*x* and *y* in arbitrary units). Three instances of *P*(*x*) are shown (uniform distributions with different means; the blue one corresponds to a mean equal to the threshold value). When the signal is always beyond the threshold (red distribution) the circuit exhibit a nonzero MI only when it works stochastically (note the output distributions). Here we considered a binary response (OFF if *y* < 1, ON otherwise). (C) Resonance in MI as a function of intrinsic noise for the red *P*(*x*) in (B). MI values computed with the responses of the device to 10^4^ different signals drawn from the described distribution. Responses are shown explicitly for three noise strengths (inset figures, black dots), together with its averaged stimulus-response profile (red curve). The maximum in MI occurs when the averaged stimulus-response profile is more linear (S1 Fig). (D) Other signal distributions, in which intrinsic noise always reduces MI, can nevertheless exhibit a resonance when the combined response of several units is considered (we show here the case of duplicated threshold devices; *N* = 2). Colors correspond to those distributions shown in (B). See Materials and Methods for details.

We examined this issue within the precise framework of information theory. Biological circuits are in this way interpreted as communication channels, in which an input signal (*x*) originates –as a result of a cellular decision– an expression output (*y*), with a given probability (Fig 1A). The uncertainty on the input signal is then reduced by the decision process, whose set of outcomes tells us about the input distribution [16, 17]. An association that is properly quantified by the mutual information (MI), an information-theoretic measure describing the dependence between the input signal and the output phenotype (no matter how they could correlate [18], Fig 1A). Notably, this framework was recently exploited to quantify the functionality of transcriptional regulatory elements [19–21], the accuracy of cell location during developmental processes [22], and the maximal information transmission capacity of noisy signaling pathways [23, 24]. The relevance of redundancies was already manifested in some of these results.

Here, we first illustrate how the stochasticity of biochemical reactions (intrinsic noise) can help to gain information. We then show that information transfer can be amplified, if the combined response of multiple genetic units is considered. The reported amplification is shown to rely on the presence of different factors that contribute to generate variability in the individual response of each unit, like intrinsic noise or genetic heterogeneity (i.e., differences in the biochemical properties). This variability helps to enlarge the capacity of the global output to represent the input distribution. In contrast, we also discuss how factors reducing variability in the responses, like a noise source common to all units (extrinsic noise) or regulatory cross-talk, eventually mitigate the gain.

## Results

### Intrinsic noise can amplify information transfer

We first analyzed a minimal regulatory circuit implemented by a gene (whose expression we denote as *y*) autoactivating transcriptionally its own production [25] (Materials and Methods). This is a genetic implementation of a threshold device that, by acting deterministically, becomes activated only if the input signal *x* crosses a particular limit (Fig 1B). When the signal is stochastic, the response depends of course on the relationship between this threshold and the mean (and variance) of the underlying distribution *P*(*x*) (considered for simplicity as a uniform distribution; Fig 1B). A symmetric distribution centered on the threshold would thus originate equally likely the two output values (OFF/ON) (i.e., one bit of information); while the same distribution centered above/below the threshold would produce biased responses (i.e., less than one bit of information).

However, the previous behavior can be affected by the extensive noise sources acting on biological circuits [1–3]. One could ask then to what extent the circuit is reliably representing the signal. With this goal, we computed the response (black dots in subpanels of Fig 1C) to a number of signals drawn from a fixed distribution (red distribution in Fig 1B) and strength of intrinsic noise. To quantify how much information the response conveys about the input, we made use of MI [16–18] (Fig 1A) (Materials and Methods). Values of MI change with noise [main plot in Fig 1C; the mean of *P*(*x*) is above the threshold]. For weak noise levels, the circuit works essentially as a deterministic switch, it is always *y* = ON as *x* > threshold. For strong noise levels, the device cannot distinguish signal fluctuations, then its behavior is essentially random. In both cases, the gene is processing a limited amount of information (subpanels of Fig 1C, red curves denote the averaged stimulus-response profiles). But the transmission of information presents a maximum for an intermediate noise level. In this regime, the circuit can express its two possible states due to noise (i.e., low values of *x* can cross the threshold) [26], what precisely contributes to a better representation of the input signal (see also S1 Fig); a characteristic behavior of noisy nonlinear systems known as stochastic resonance (SR) [27, 28].

Moreover, SR disappears when the mean of *P*(*x*) is close to the threshold, as stochasticity is now not required to reach the two possible states. Noise always reduces information transfer (Fig 1D, curves for *N* = 1, *N* denoting the number of circuits involved). Note here how MI does exhibit an upper limit of one bit when the mean of *P*(*x*) exactly matches the threshold, and the circuit is noiseless. MI decreases with noise because signal values above/below the threshold originate in some cases stochastic crossings (e.g., *y* = ON when *x* < threshold), and the information content in absence of noise is already high (note in contrast that, in the scenario of SR, MI was very low in absence of noise). Additionally, Fig 1D displays a situation in which a maximum in MI is nevertheless observed (curves for *N* = 2). This is obtained by increasing the number of devices processing the same input, with *y* representing the sum of all individual outputs; a phenomenon called suprathreshold SR [29] (see S1 Text, and S2 Fig, for a brief account of suprathreshold SR). Note how the maximum is observed in the noise regime where the information processing of individual units (*N* = 1) qualitatively declines (from here the presence of several units begins to lose effectiveness). What is apparent is that redundancy boosts information transfer, given a fixed noise level.

### Genetic redundancy enhances information transfer

**Fig 2:**
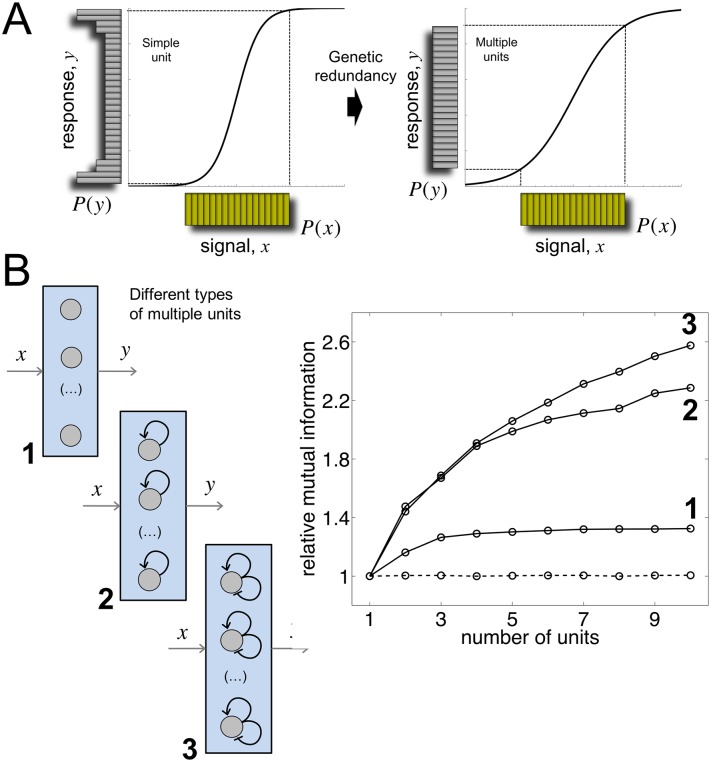
Genetic redundancy amplifies information transfer in threshold genetic systems. (A) Input/output distributions depicting information transfer. The input distribution (in yellow) is assumed to be uniform. Output distributions (in gray) illustrate the processing of the signal *x*, either through a single copy of the threshold device (left) or an array of multiple redundant copies (right). In the latter case, each unit of the array receives the same signal and the output *y* is the sum of all the individual responses (S2 Fig). Redundancy enlarges the alphabet of the response. This is reflected in the output distribution, and also in the linearization of the averaged stimulus-response profile (black curve). (B) (Left) Array of *N* threshold devices (circles) whose constituent units correspond to (1) a simple regulated unit, (2) a bistable circuit implemented with a positive feedback, and (3) an excitable circuit constituted by two interlinked positive and negative feedback loops. (Right) Dependence of mutual information (MI) with the number of units (*N*) for each of these systems relative to the case *N* = 1. A uniform signal distribution with mean equal to the threshold value was considered. MI does not increase with extra copies for noiseless units (independently of the type of unit; dashed line). See Materials and Methods for details on the modeling of each circuit.

The addition of extra copies of the threshold device, i.e., genetic redundancy, appears then as a potential mechanism to increase the transmission of information in the presence of intrinsic noise. Consider, for instance, a situation in which two devices read in parallel the same input signal, assuming again two possible values of gene expression for each unit. The overall output *alphabet* [30] consists of three *letters*: {0 (both copies OFF), 1 (one OFF the other ON), 2 (both ON)}. The new alphabet is linked, of course, to the action of independent (intrinsic) noise sources acting on the two genes, which allows each device to produce an autonomous response (with noise-induced threshold crossings, S2 Fig). The sum of individual responses would give, accordingly, a global output distribution *P*(*y*) constituted by three peaks. The extended alphabet helps therefore to enlarge the capacity of the output to represent the input variability; in other words, it contributes to linearize the averaged stimulus-response profile (Fig 2A, see also S3 Fig).

Both the number of units and the type of nonlinearity influence the increment of information transfer. In Fig 2B, we introduced three different threshold devices [25] to show how MI increases with redundancy. For each type, MI relative to the case of no redundancy (i.e., a single unit) was plotted. Specifically, we examined a simple regulated unit, a bistable expression system implemented through a positive feedback, and an excitable device constituted by interlinked positive and negative feedbacks (implemented as the one linked to transient differentiation in *Bacillus subtilis* [8]) (Materials and Methods). The output of all these devices is given by a continuous variable representing gene expression (note that the response of the bistable unit was regarded as OFF/ON in the previous section, Materials and Methods). This allowed identifying discrepancies in terms of MI among different gene regulatory circuits. In particular, the largest amplification of information content corresponds to those devices whose actions ultimately rely on discontinuous transitions (i.e., the bistable and excitable systems). Out of these two systems, the excitable one presents comparatively larger amplification, although only observed for relatively large arrays. In this system, the response is entirely binary even in presence of noise: either the signal triggers a response or not (S4 Fig). Moreover, the gain in information transfer is much lower for the simple regulated system in which the response profile is continuous (what entails that one unit already has the capacity to reach a relatively large output alphabet). The contribution of redundancy is therefore always much higher in analog-to-digital than in analog-to-analog signaling circuits, and provided they are noisy.

### Input signal distribution shapes information transfer

**Fig 3:**
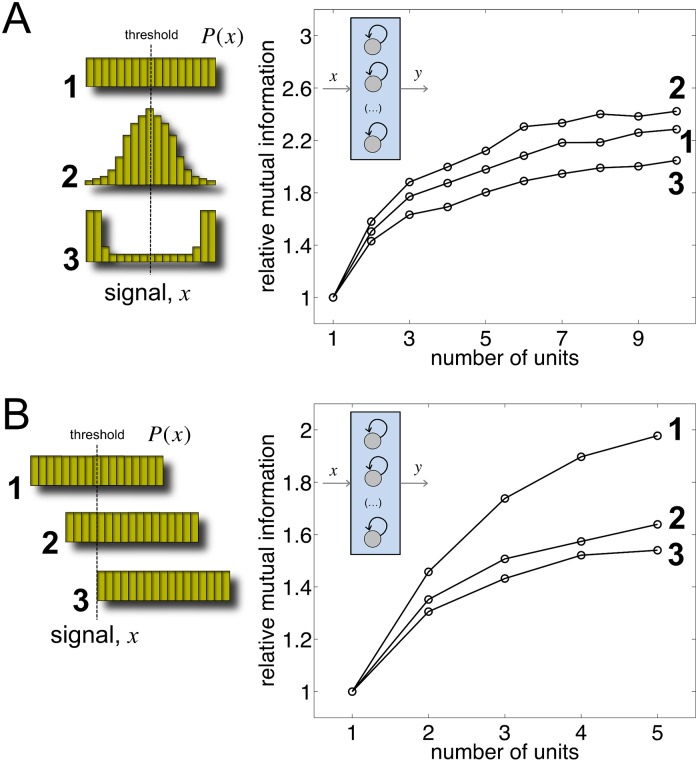
The distribution of the signal modulates the increase of information transfer due to genetic redundancy. (A) Effect of the form of the distribution on MI: (1) uniform (covering two orders of magnitude), (2) lognormal (with standard deviation equal to 2/3), and (3) beta in log scale (with the two shape parameters equal to 1/3). In all cases, the mean of the distribution is equal to the threshold value. (B) Effect of the mean of the distribution (here uniform) on MI: (1) equal to the threshold value, (2) and (3) deviated from the threshold value. We considered as threshold device a bistable unit implemented with a positive feedback in all plots (Materials and Methods).

We next studied how the specific distribution of the signal impinging on the genetic circuits (that can encode distinct environmental or genetic conditions [31]) can further modulate the enhancement of information transfer. Knowing this distribution is typically difficult in cellular systems [24]. We examined then to what extent information transfer would be influenced by the shape of *P*(*x*). We considered three different signals acting on the array of threshold devices (Materials and Methods). MI increases more strongly with a normally distributed signal (Fig 3A). For this distribution, the mass of *x* values is closer to the threshold what allows noise to alter more frequently the expected deterministic response of the device. We then analyzed the effect of the relationship between the threshold and the signal mean. When the mean of *P*(*x*) is equal to the threshold, a higher increase of MI with genetic redundancy is observed (Fig 3B). Arguably, if the mass of *x* values is equally distributed above/below the threshold, there exists again more chances for noise-induced threshold crossings. Fine-tuning of the parameters characterizing *P*(*x*) contributes thus to a better representation of the input signal by the global output response.

### Extrinsic noise and cross-talk limit information transfer

**Fig 4:**
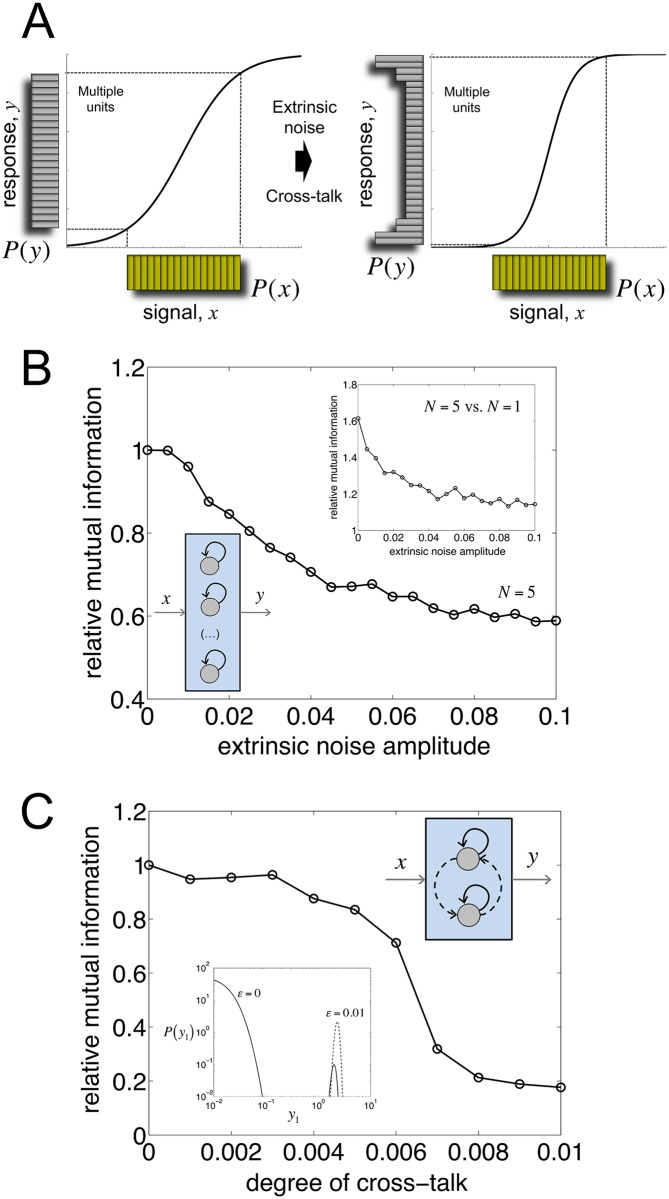
Extrinsic noise and cross-talk among redundant copies limit information transfer. (A) Input/output distributions depicting information transfer. Correlation among individual gene responses due to extrinsic noise or cross-talk reduces the response alphabet, and generates a less linear averaged stimulus-response profile (black curve, see Fig 2A for comparison). (B) Dependence of mutual information (MI) with the strength of extrinsic noise (Materials and Methods). Relative MI is with respect to absence of extrinsic noise. For this plot, we considered a system of *N* = 5 bistable units implemented with positive feedback. The inset shows a direct comparison between *N* = 1 and *N* = 5, emphasizing that MI increases with *N*. (C) Dependence of MI with the degree of cross-talk for the same regulatory system, but now constituted by *N* = 2 units. Relative MI is with respect to the situation without cross-talk. The inset presents the marginal probability distribution of gene expression of one unit (*y*_1_) in the absence and presence of cross-talk (parameterized by *ε* = 0 and *ε* = 0.01, respectively; see Materials and Methods) for the mean value of the input signal (*x*). Note that when the units are coupled, gene expression becomes unimodal (dashed curve).

The most important constraint for the gain in information associated to the previous redundant systems is the independence between the noise sources. When these are correlated, *P*(*y*) becomes more sharply peaked around a small subset of possible responses (i.e., the output alphabet is more limited; Fig 4A). This applies to biological circuits that, in addition to intrinsic noise, also integrate the effect of extrinsic fluctuations [2, 32]. Extrinsic noise affects all genetic devices in the same manner what eventually correlates individual outputs. For instance, Fig 4B shows how (relative) MI decreases with the strength of extrinsic noise in an array of five bistable units (Materials and Methods). Note however that this redundant architecture still exhibits, for different extrinsic noise levels, a larger MI with respect to the nonredundant case (inset of Fig 4B).

Despite the independence of the noise sources, cross-talks between devices can similarly lead to correlations in the individual responses. In a genetic context, one could imagine two independent transcription factors sharing recognition domains [33]. One could also conceive a second unit recently emerged by duplication, and that no process of neofunctionalization yet occurred [34]. Fig 4C indeed shows a decay in (relative) MI for a system of two bistable units when cross-talk between them increases (simulations done without accounting for extrinsic noise, Materials and Methods, see also S5 Fig for a study of asymmetric cross-talk). In this case, the activation of one unit drags the activation of the other, biasing again the output alphabet (inset of Fig 4C). Of note, the decay profile in MI is qualitatively different in the two scenarios. Addition of extrinsic noise contributes to limit information transfer in a progressive manner since it increasingly coordinates responses. In the second situation, outputs are correlated once a certain cross-talk range is reached what is reflected in a sharper decay (similar patterns are expected to be observed when considering other constituent units).

### Heterogeneity also contributes to increase information transfer

We proceeded by examining a complementary source of individuality in information processing, which is linked to the heterogeneity within the collection of threshold devices. In the context of genetic circuits, variability in promoter strengths, ribosome-binding sites, proteins half-lives, or protein-DNA binding affinities are all factors that in effect modify threshold values or output responses. Adjusting for each device the values of the biochemical parameters of the model can capture this variation [35]. We specifically explored the implication of threshold heterogeneity in the array of five bistable units in which the threshold values are drawn from a Gaussian distribution (and heterogeneity equates to the associated standard deviation, Materials and Methods).

**Fig 5:**
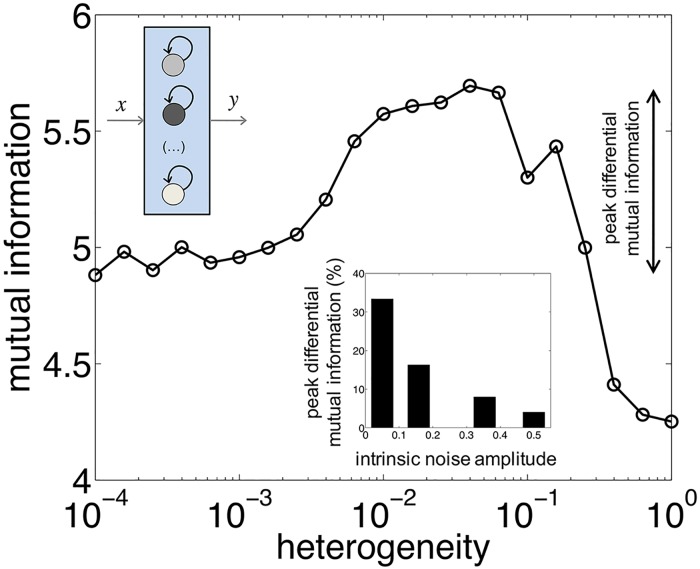
Genetic heterogeneity among redundant copies amplifies information transfer. Variation in the biochemical features of the constituent threshold devices (represented by the different colors of the devices, here bistable units, *N* = 5) leads to a maximum in mutual information (MI). We consider random threshold values drawn from a Gaussian distribution whose standard deviation determines the degree of heterogeneity (see Materials and Methods, no heterogeneity corresponds to a very low, i.e., 10^−4^, but nonzero value due the log scale of the *x* axis). The inset indicates the peak differential MI (i.e., the difference between the largest value of MI with heterogeneity and the value of MI without it) for varying noise levels. This reveals how a situation of stronger intrinsic noise contributes to reduce the improving effect on MI of heterogeneous units (the main plot corresponds to an intrinsic noise amplitude equal to 0.16).

Notably, we observed again a resonance in information transfer, but this time as a function of the degree of heterogeneity (Fig 5). While moderate levels of heterogeneity allows regulatory circuits to encode complementary aspects of the input signal, hence enhancing information transfer, greater variation becomes detrimental by originating noise-induced threshold crossings over the whole input range. Moreover, since both intrinsic noise and heterogeneity contribute to increase the transmission of information (certainly by enlarging the alphabet of the global output), we also explored to what extent these two sources of individuality act independently [36]. We found that intrinsic noise mitigates the increase in MI due to threshold variability (inset of Fig 5). Intuitively, higher noise levels make indistinguishable those variations associated to the control of gene expression in the threshold unit.

### Empirical evidences of the theory

Several analyses support the gain in information transfer established by our theoretical framework. For instance, by explicitly quantifying information transduction, new experimental reports showed how multiple copies of a biochemical system accessing the same signal leads to an increase in MI [23, 24]. This was coupled to multiple gene copies (comparing 1× and 2× diploids [24]) or to a general redundant architecture (with a bush network topology) that could be implemented in an intra- or intercellular manner [23].

**Fig 6:**
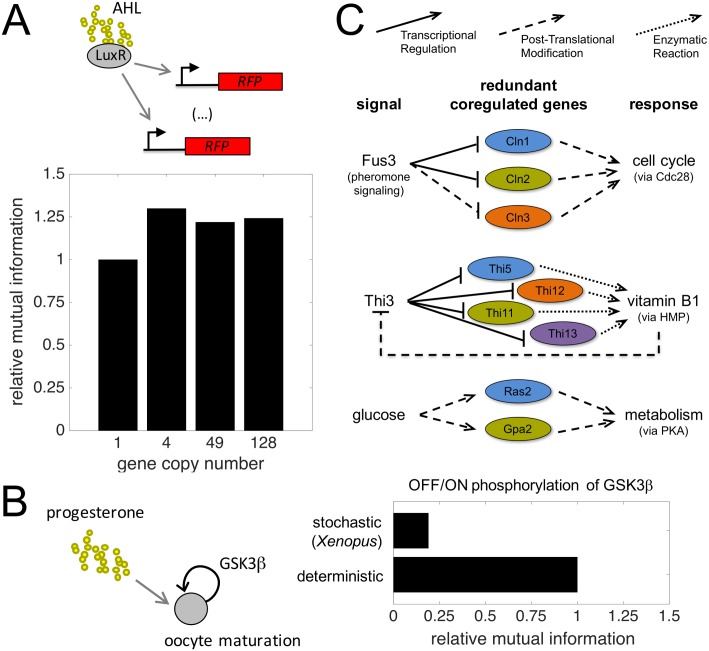
Experimental evidence of the role of genetic redundancies for the transmission of information. (A) (Top) Regulatory scheme of a synthetic experimental system taken to assess the effect of genetic redundancy on information transfer. An array of *N* RFPs regulated by LuxR-AHL, implemented through chromosomal integration (*N* = 1) or plasmids with different copy numbers (*N* > 1). (Bottom) Dependence of mutual information (MI) with the number of units (*N*) relative to the system with *N* = 1 (Materials and Methods). (B) (Left) Regulatory scheme associated to the induction of oocyte maturation in a bistable manner (mediated by *GSK*3*β*) by progesterone. (Right) Effect of molecular noise on information transfer. MI for this intrinsically stochastic threshold unit relative to the deterministic case (Materials and Methods). (C) Three examples of genetic regulatory architectures in yeast where redundant genes are coregulated by a common signal. They correspond to pheromone, vitamin B1 and glucose signaling, respectively.

Moreover, recent work examined how the output of a LuxR-inducible promoter in *Escherichia coli* would change with copy number ranging from 1 copy per cell (genome copy) to hundreds (via multicopy plasmids) [37]. We used the available data to confirm the relative MI gain as a function of genetic redundancy (Fig 6A, Materials and Methods). Because the system does not rely on a discontinuous transition (e.g., it is not bistable), the gain saturates with few copies (see the curve labelled as “1” in Fig 2B). We contrasted this analysis with the response to progesterone of a single bistable unit governing the oocyte maturation in *Xenopus* [38]. The noise-driven differential concentration from oocyte to oocyte (for a particular progesterone level) of the controlling protein makes unreliable the maturation process (i.e., subthreshold levels of progesterone can induce maturation in some oocytes) what limits the amount of information transmission one could achieve in a noiseless situation (Fig 6B). Arguably, the presence of additional copies of the precursor gene would help to reduce such variability and then mitigate the loss of information (see Fig 1D).

Finally, Fig 6C illustrates other biological contexts in which the signaling architectures, by exhibiting redundancies, could be more effective in terms of information transfer following our predictions [39–41].

## Discussion

Binary decisions implemented by means of threshold devices appear in many engineering and physical systems, and have been extensively studied in relation to the detection and transmission of signals. While noise was commonly considered harmful in many of these settings, some work alternatively identified circumstances in which its presence improves performance [27, 28, 42]. In Biology, both the stochastic nature of biochemical reactions and the typical occurrence of thresholds –linked, for instance, to cell fate determination– also anticipates the possibility of beneficial effects. This specifically applies to the case of gene regulatory circuits, in which molecular stochasticity acts in many cases as a core determinant of function [43].

**Fig 7:**
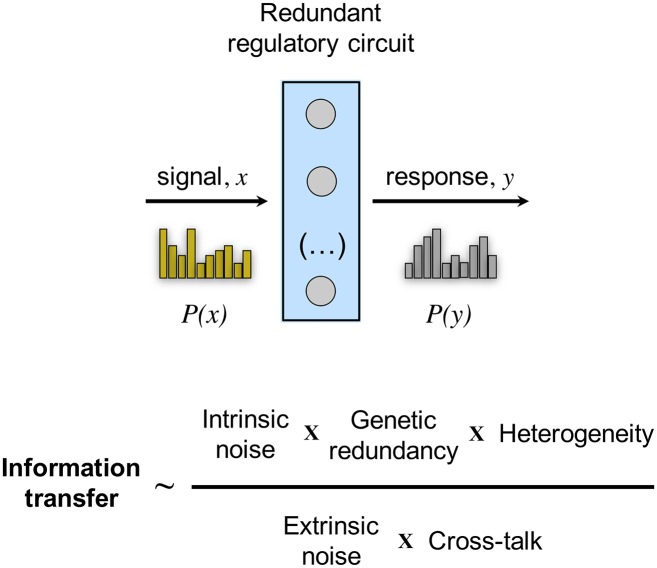
Model of information transfer in gene regulatory circuits. Intrinsic noise, genetic redundancy, and heterogeneity increase the transmission of information by expanding the capacity of the (summing) global output to represent the input variability. In contrast, extrinsic noise and cross-talk among redundant units become limiting factors by correlating the individual outputs of the units. Threshold genetic units represented here as gray circles; input signal *x* and output response *y* characterized by *P*(*x*) and *P*(*y*) distributions, respectively.

In this work we discussed in detail the benefits of intrinsic molecular noise when multiple threshold regulatory circuits process a common signal. This system exhibits a resonance phenomenon known as suprathreshold SR [29] (S1 Text). The effect establishes the value of the noise-induced uncoupling of the action of each unit. This advantage is manifested as well in a more linear relation between stimulus and response, a type of dose-response alignment that could be important in how precise extracellular conditions determine cell responses, and that was previously associated to negative feedbacks [21]. Our functional analysis therefore reveals redundancies not only as a genetic architecture contributing to robustness [44, 45], or to the adaptation to novel environments through the increase of gene expression levels [46, 47], but also as a mechanism increasing the capacity to transmit reliable information (Fig 7). We suggest that this aspect could contribute to the evolutionary maintenance of genetic redundancy (tradeoffs with the associated genetic load of redundancy could matter, see S6 Fig). That multiple signaling pathways in *Saccharomyces cerevisiae* overlap supports this hypothesis [48].

The balance of intrinsic/extrinsic noise also plays an important part to condition the amount of information transferred (Fig 7). Cells implementing regulatory circuits with few representative molecules or living in rich environments would shift this balance towards intrinsic noise [49]. Beyond this genetic/environmental tuning, cellular systems could avoid the loss of information, due to extrinsic noise, when the signal operates dynamically rather than statically [50]. Note that here we considered a static operation. Our results further emphasize how heterogeneity and cross-talk among redundant copies play opposite roles in the maintenance of information content (Fig 7). One could thus interpret the action of several parallel signaling pathways, each conveying approximately one bit of information, as heterogeneous copies of an effective threshold device what enhances information transmission, e.g., this was observed in pathways for the growth factor-mediated gene expression [51].

That a global response –the sum of individual responses, in this case– implemented by parallel processing units could lead to better performance than that of the individual components was proposed in early models of computing, and can indeed be observed at different levels of biological organization: from genes (this work), to living cells [23], to social organisms [52]. In addition, ideas on redundancy and heterogeneity when mounting unreliable components were already present in the initial development of fault-tolerant computation and communication [53, 54], and also permeate to many biological scenarios. Our work substantiates the implications of these notions in cellular decision making by natural [48] and synthetic [55] molecular circuits, and contributes to exemplify how the application of concepts from information theory could lead to a more precise and quantitative understanding of cellular systems.

## Materials and Methods

### Modeling noisy regulatory systems

As a general regulatory model we examined a redundant system consisting of *N* different units, each of them activated by the input signal (*x*). These units correspond to three types, defined by three specific sets of ordinary differential equations that were extended to account for stochastic effects using the Langevin approach [56].

#### Simple regulated system

The model for the *i*-th unit of this type is

$$\frac{dy_{i}}{dt}=\alpha_{0}+\frac{\alpha x{\left(t\right)}^{n}}{1+x{\left(t\right)}^{n}}-y_{i}+q_{i}\left(y_{i},x\right)\xi_{i}\left(t\right),$$ (1)

where expression, and time, are appropriately rescaled to have a dimensionless model. Here, *α*_0_ and *α* correspond to the basal and maximal expression level, and *n* governs the steepness of the function. The parameter values used are *α*_0_ = 0.01, *α* = 2.5, and *n* = 2. Intrinsic noise is described by an stochastic process *ξ*_*i*_ with 〈*ξ*_*i*_(*t*)〉 = 0 and 〈*ξ*_*i*_(*t*_0_)*ξ*_*i*_(*t*_0_ + *t*)〉 = *δ*(*t*). Noise amplitude is given by $q_{i}\left(y_{i},x\right)=\sqrt{\frac{1}{K}(\alpha_{0}+\frac{\alpha x^{n}}{1+x^{n}}+y_{i})}$, i.e., the square root of the sum of the propensities [56]. *K* is proportional to the effective dissociation constant between the transcription factor and the promoter, and determines the number of molecules of the system and then intrinsic noise [57] (*K* = 100, otherwise specified).

#### Bistable system

We considered a gene activating transcriptionally its own expression. This corresponds to a minimal implementation of a bistable system. The model for the *i*-th unit reads

$$\frac{dy_{i}}{dt}=\alpha_{0}+\frac{\alpha y_{i}^{n}}{1+y_{i}^{n}}-y_{i}+x\left(t\right)+q_{i}\left(y_{i}\right)\xi_{i}\left(t\right),$$ (2)

where expression and time are again appropriately rescaled to have a dimensionless model. Parameters definitions and values as before, as well as the statistics of *ξ*_*i*_. The input signal (*x*) is introduced in this case as a small perturbation. Noise amplitude is given by $q_{i}\left(y_{i}\right)=\sqrt{\frac{1}{K}(\alpha_{0}+\frac{\alpha y_{i}^{n}}{1+y_{i}^{n}}+y_{i})}$, having neglected the effect of *x*.

#### Excitable system

We used a model previously proposed to explain competence in *Bacillus subtilis*, associated with the capability for DNA uptake from the environment, by which the cell can reach transient (excitable) differentiation [58]. Each of the *N* units of the system consists of two transcriptional units (*y*_*i*_ and *z*_*i*_) that implement, in an effective way, interlinked positive and negative feedback loops. The model for the *i*-th unit reads

$$\begin{array}{cc} \frac{dy_{i}}{dt} & =\alpha_{0}+\frac{\alpha {\left(\sigma y_{i}\right)}^{n}}{1+{\left(\sigma y_{i}\right)}^{n}}-\frac{y_{i}}{1+y_{i}+z_{i}},\\ \frac{dz_{i}}{dt} & =\frac{\beta }{1+{\left(\sigma y_{i}\right)}^{m}}-\frac{z_{i}}{1+y_{i}+z_{i}}+x\left(t\right)+q\xi_{i}\left(t\right), \end{array}$$ (3)

where expression and time are appropriately rescaled to have a dimensionless model. The parameter values are *α*_0_ = 0.004, *α* = 0.07, *β* = 0.826, *σ* = 5, *n* = 2, and *m* = 5 (*β* and *m* correspond to maximal expression level and steepness, respectively, while *σ* describes a ratio of dissociation constants). Here, noise amplitude is constant ($q=\sqrt{(\beta +1)/K}$, with *K* = 500), and *ξ*_*i*_ follows the same statistics as before.

### Extrinsic noise, cross-talk and heterogeneity

We introduced an additional stochastic process *ξ*_*ex*_, to account for extrinsic noise common to all units. The correlation time of extrinsic noise is of the order of the cell cycle (the mean is also 0). For simplicity, we assumed systems implemented with short-lived proteins, so that *ξ*_*ex*_ is constant within the time window required for the dynamical unit to reach steady state after reading the signal *x* (this feature also reduces potential expression dependences on growth rates, e.g., [59]). To examine cross-talk we applied a perturbative approach, with a perturbative parameter *ε* quantifying the degree of cross-talk. Finally, to study heterogeneity we specifically considered variability in the threshold values of the different units. We modified these values by introducing a Gaussian random number *ω* (of mean 1), with its standard deviation corresponding to the degree of heterogeneity. Note that only intrinsic noise was considered when accounting for cross-talk or heterogeneity. See full details of these methods in the S1 Text.

### Input and output variables

We considered that the threshold regulatory system is initially in a steady state (*x* = 0) before becoming activated (*x* ≠ 0 at time *t* = 0). The signal represents a continuous stimulus with fixed amplitude (*x* is a step function at *t* = 0), for the simple and bistable units, or a pulse (for one unit of normalized time) for the excitable one. The amplitude of the signal is given by *x* = 〈*x*〉10^*u*^, where *u* corresponds to a random number uniformly distributed in [−1, +1], unless otherwise specified. Signal stochasticity illustrates fluctuations due to upstream processes, environmental changes or molecular noise. We considered log*x* as input variable to compute information transfer.

Each threshold unit is able to sense the signal what could alter its expression level as Δ*y*_*i*_ = *y*_*i*_(*x*) − *y*_*i*_(*x* = 0). The output was calculated at steady state, and signal fluctuations occur at a frequency that allows the genetic circuit to respond against the current signal value. In addition, the total differential gene expression of a redundant system can be written as $\Delta y=\sum_{i=1}^{N}\Delta y_{i}$. Since the response of the excitable system is transient, we implemented a Boolean function operating on *y*_*i*_, setting 1 if the unit was excited or 0 if not. For all main figures, we always treated the threshold units as dynamical systems, i.e., modeled by differential equations. However, in the first section of the paper (Fig 1), the gene expression level (*y*_*i*_) was treated as a Boolean variable (OFF/ON) after resolving the corresponding differential equation. Expression was treated as a continuous variable in the subsequent sections (Figs 2–5).

### Input distributions

We mainly included a uniform distribution *P*(*x*) covering two orders of magnitude throughout the manuscript (as described above). However, in Figs 1 and 3B, we analyzed the effect of the mean 〈*x*〉 of the distribution, with values 0.001 (equal to the threshold value), 0.005 and 0.01. In Fig 2, the mean was fixed to the threshold value, i.e., 〈*x*〉 = 1 in the simple regulated unit, 〈*x*〉 = 0.001 in the bistable system, and 〈*x*〉 = 0.9 in the excitable system. In Figs 4 and 5, concerning to the bistable system, 〈*x*〉 = 0.005. Moreover, in Fig 3A, we studied the effects of a normal or a beta distribution in log scale, with the mean equal to the threshold value.

### Quantification of information transfer

We used mutual information (I) as a quantitative metric to describe how the global output response of a single cell is sensitive to different concentrations of the input signal [18]. This adds to the quantification by the averaged stimulus-response profile. To calculate I, we performed 10^4^ realizations of the pair (*x*, *y*) and solved numerically the following integral

$$I=-\int_{-\infty }^{+\infty }P_{\Delta y}\left(s\right)\log_{2}P_{\Delta y}\left(s\right)ds+\int_{-\infty }^{+\infty }P_{\log x}\left(r\right)\times \int_{-\infty }^{+\infty }P_{\Delta y|\log x}\left(s\right)\log_{2}P_{\Delta y|\log x}\left(s\right)dsdr,$$ (4)

where we considered log*x* as input and Δ*y* as output variables. By using the Fokker-Planck equation, we calculated the probability that a unit has a given gene expression level (see more details in S1 Text).

### Analysis of experimental data

We considered the dose-response data of a synthetic system composed by a red fluorescent protein (RFP) controlled by the transcription factor LuxR, which is activated by N-acyl homoserine lactone (AHL, the signal) [37]. Indeed, this is a simple regulated unit, which was implemented with different gene copy numbers. Mutual information was calculated between the RFP expression at the population level and the concentration of AHL in log scale (an estimation of the actual values). In addition, we considered the dose-response data of a natural system governing the oocyte maturation in *Xenopus* [38]. Here, the glycogen synthase kinase 3*β* (*GSK*3*β*) controls the meiotic entry of progesterone (the signal) in the oocytes. This system is bistable and is implemented by an effective positive feedback loop (through two negative regulations). Mutual information was calculated between the phosphorylation state of *GSK*3*β* of individual oocytes (considered as a Boolean variable) and the concentration of progesterone in log scale. As a reference, we considered a deterministic scenario with a distribution of progesterone centered in the threshold of the system.

## Supporting Information

S1 Text A detailed description of all the methods used in this study. (PDF)

S1 Fig Distance of the average response to the ideal linear response as a function of intrinsic noise. We computed the change in gene expression Δ*y* (with respect to the initial steady state of the system) caused by the presence of the signal (*x*) for the bistable unit (*N* = 1). The distribution of the input signal values is a uniform with a mean of 0.005 and a variance that allows covering two orders of magnitude. Panels correspond to the responses of the device to 10^4^ signal values drawn from the described distribution (black dots) and different intrinsic noise amplitudes. Average response (red curve, mean over all possible responses) and ideal linear response (blue curve) are also shown. The main plot displays the distance between the average and the linear response, which exhibits a minimum for certain amount of noise.
(TIF)

S2 FigSuprathreshold stochastic resonance in multilevel threshold systems.(A) A signal *x* acting on a summing array of threshold units *i* (threshold value *θ*_*i*_), each one experiencing independent noise *ξ*_*i*_. The output *y* ranges from 0 to *N* (the total number of units). (B) Average transmitted information through the array in (A) quantified in terms of the mutual information (I). Here, 10^4^ signal values, drawn from a Gaussian distribution with mean 〈*x*〉 = 1 and *σ*_*x*_ = 1, passed through the threshold device (Heaviside function). All thresholds are set equal to the signal mean, and noise strength is determined by *σ*_*i*_ (of a Gaussian distribution with 〈*ξ*_*i*_〉 = 0). In this situation, the signal is strongly suprathreshold, yet noise does induce a maximum (stochastic resonance) for all *N* > 1. (C) Quantification of information transmission using the correlation coefficient cannot distinguish between two situations with low (*r*_1_) and high (*r*_2_) noise strength as compared to the mutual information ($I_{1}$ and $I_{2}$, respectively). Here, we considered an array of *N* = 5 threshold units. (D) Response (blue) and averaged response (red) in the regimes of low and high noise in (C); values beyond the threshold highlighted in yellow. While the linear dependence between *x* and *y* is relatively similar, the information obtained about *x* when measuring *y* is not. This implies similar correlation coefficients but different mutual information. The use of I to quantify the amount of information transmission was originally discussed in [29].(TIF)

S3 Fig Effect of feedback strength or intrinsic noise on information transmission. (A) Dependence of mutual information with the feedback strength (or also intrinsic noise amplitude, parameterized by 1/*K*) of the bistable unit. The inset shows the effective stochastic potential (*ϕ*) for *K* = 100 and *K* = 1000 (with *N* = 2). Certainly, it shows two potential wells (i.e., two stable steady states), and the threshold of the system is within (note that intrinsic noise is multiplicative, i.e., the amplitude of the stochastic fluctuations depends on the particular gene expression level). In case of higher noise (*K* = 100), the potential barrier is very low, indicating that it is very easy to have stochastic threshold crossings. However, in case of lower noise (*K* = 1000), the potential barrier is high, moderating the number of stochastic threshold crossings. The observed trend of information transfer versus intrinsic noise (higher the noise, higher the information transfer) is explained because the stochastic threshold crossings (for a continuous output variable, and to some extent) is the mechanism underlying the linearization of the response and the increase of communication fidelity. (B) Dependence of mutual information with the number of units (*N*) of the system. Relative mutual information is with respect to *N* = 1 for different intrinsic noise levels (modulated by *K*). This shows how the higher the intrinsic noise level, the stronger the amplification of information transfer due to genetic redundancy.
(TIF)

S4 Fig Influence of the number of units for an excitable system. (Top) Input/output distributions depicting information transfer. The input distribution (in yellow) is assumed to be uniform (with a mean of 0.9 and a variance that allows covering two orders of magnitude). Output distributions (in gray) illustrate the processing of the signal *x*, either through a single copy of the threshold device (left) or an array of multiple redundant copies (right). Note that the output is Boolean, setting if the unit is excited or 0 if not. In the latter case, each unit of the array receives the same signal and the output *y* is the sum of all the individual responses. Redundancy effectively enlarges the alphabet of the response. This is reflected in the output distribution, and also in the linearization of the averaged stimulus-response profile (black curve). (Bottom) Relative mutual information, with respect to *N* = 1, for a system of *N* excitable units implemented with interlinked positive and negative feedbacks. According to the same input level, and in the presence of molecular noise, each unit can perform an excursion over the phase space (excitation) or not. Inset shows the phase space where the nullclines (black lines) determine the possible dynamical trajectories (gray lines, deterministic regime). Arrows indicate direction of the dynamics, and the black dot corresponds to a stable steady state. Note that a perturbation (in gene *z*) induces the excitation of the system provided its magnitude (*x*) is large enough, otherwise the system falls down to the steady state as it remains within the corresponding basin of attraction.
(TIF)

S5 Fig Dependence of mutual information with the average degree of cross-talk for the bistable system constituted by 2 units. We considered a scenario of asymmetric cross-talk (parameterized by *ε*), i.e., gene 1 is affected by gene 2 with strength *ε*_2_ and gene 2 by gene 1 with strength *ε*_1_, with *ε* = (*ε*_1_ + *ε*_2_)/2. Note that the average amount of cross-talk is maintained with respect to a scenario of symmetric cross-talk. Three situations are represented (black, *ε*_1_ = *ε*_2_ = *ε*, symmetric; blue, *ε*_1_ = 1.5*ε* and *ε*_2_ = 0.5*ε*; and red, *ε*_1_ = 2*ε* and *ε*_2_ = 0), all displaying a similar trend.
(TIF)

S6 Fig Tradeoff between information transfer and genetic load for a bistable genetic system with redundancy. Simulations with *N* ranging from 1 to 10 (genetic load). For simplicity, we considered a bacterium of 5000 genes. According to previous work characterizing genetic load in bacteria [60], we can state that the relative growth rate of the cell is 1 − *ϕ*/0.48, where *ϕ* is the relative amount of unnecessary protein. In our case, *ϕ* = *S*(*N* − 1)/5000, where *S* is the number of independent systems that are implemented with redundancy. In case of a specific strategy by one system (*S* = 1), the genetic load is negligible. However, if redundant systems were more generally observed (e.g., *S* = 100), the genetic load would be considerable what leads to a Pareto front.
(TIF)
