# Supplementary material for: Genetic Redundancies Enhance Information Transfer in Noisy Regulatory Circuits
Source: PLoS Comput Biol. 2016 Oct 14;12(10):e1005156. doi: 10.1371/journal.pcbi.1005156 (PMC5065233; doi:10.1371/journal.pcbi.1005156)
Supplement: S1 Text — (PDF) [file pcbi.1005156.s001.pdf]

Supplementary Material

Genetic redundancies enhance information transfer in noisy regulatory circuits

Guillermo Rodrigo[1] and Juan F. Poyatos[2]†

[1] Instituto de Biología Molecular y Celular de Plantas, CSIC–UPV, 46022 Valencia, Spain, [2] Logic of Genomic Systems Laboratory (CNB-CSIC), 28049 Madrid, Spain.
† Corresponding author. Logic of Genomic Systems Laboratory (CNB–CSIC), Darwin 3, Campus de Cantoblanco, 28049 Madrid, Spain. E-mail: jpoyatos@cnb.csic.es.

Supplementary Methods

Suprathreshold stochastic resonance

Suprathreshold stochastic resonance is a class of stochastic resonance phenomenon that explains how the transmission of information by a summing network of N identical nonlinear devices can be enhanced in the presence of noise. In the original work [1], each nonlinear device denotes a threshold unit, which is subject to the same input signal $x(t)$ but independent Gaussian noise $\xi_i(t)$, with same standard deviation (Fig. S2A). Each unit i is modeled as a Heaviside function (with threshold level θ_i), and each output, $y_i(t)$, is equal to unity if $x(t) + \xi_i(t) > \theta_i$ (being zero otherwise). This architecture was similar in spirit to earlier work on the signal-processing activity of neuronal ensembles (the presence of a threshold represents the "firing" of a neuron). Finally, the response of the network $y(t)$ is obtained by summing the individual responses of each device, i.e., it represents the number of devices that are triggered.

The information processing capabilities of this system was measured by the mutual information $\mathcal{I}(x, y) = -\int\int P(x, y) \log_2 (\frac{P(x, y)}{P(x)P(y)}) dxdy$, where $P(x, y)$ in the joint probability distribution of input and output, and $P(x)$ and $P(y)$ are the corresponding marginal probabilities ($\mathcal{I}$ is measured in bits). Noisier input would generally lead then to lower $\mathcal{I}$, however there are situations in which performance is enhanced by the presence of a certain amount of noise (when there exists several copies of the device, Fig. S2B). Note that the use of other typical measures of dependence, like the correlation coefficient, would be less sensitive. To examine this issue, we computed the transmitted information of an input signal as a function of noise strength using either the correlation coefficient, r, or the mutual information $\mathcal{I}$ (for an array of $N = 5$ threshold units). Notably, the correlation coefficient is not strongly affected by noise strength, while the mutual information changes and exhibits a maximum, i.e., two explicit regimes of low/high noise present similar correlation coefficient $r_1 \sim r_2$ but different mutual information $\mathcal{I}_1 > \mathcal{I}_2$ (Figs. S2C-D). Since this resonance phenomenon was shown to appear regardless of whether the signal is entirely subthreshold or not the term suprathreshold was considered, to distinguish it from more standard stochastic resonance phenomena in single threshold devices (where suprathreshold signals do not experience resonance).

Extrinsic noise, cross-talk and heterogeneity

We considered the architecture of N bistable units to specifically characterize the effects of extrinsic noise, cross-talk, and heterogeneity on information transfer. Recall that in this case each i-th unit corresponds to a minimal implementation of a bistable system as

$$\frac{dy_i}{dt} = \alpha_0 + \frac{\alpha y_i^n}{1 + y_i^n} - y_i + x(t) + q_i(y_i)\xi_i(t), \tag{1}$$

where expression and time are appropriately rescaled to have a dimensionless model. The parameter values as well as the amplitude and statistics of ξ_i as described in main text (Materials and Methods).

To account for extrinsic noise, we introduced a new stochastic process (ξ_{ex}), common to all units, in Eq. (1) as

$$\frac{dy_i}{dt} = \alpha_0 + \frac{\alpha y_i^n}{1 + y_i^n} - y_i + x(t) + q_i(y_i)\xi_i(t) + q_{ex}\xi_{ex}(t). \tag{2}$$

The correlation time of extrinsic noise is of the order of the cell cycle [2] (the mean is 0). For simplicity, we assumed a system implemented with short-lived proteins, so we can assume that ξ_{ex} is constant within the time window that the unit needs to reach its steady state upon receiving the perturbation x.

To account for heterogeneity in the different units of the system, we followed

$$\frac{dy_i}{dt} = \alpha_0 + \frac{\alpha(\omega_i y_i)^n}{1 + (\omega_i y_i)^n} - y_i + x(t) + q_i(y_i)\xi_i(t), \tag{3}$$

where the standard deviation of ω_i (a Gaussian random number with mean 1) quantifies the degree of heterogeneity.

To account for certain cross-talk between the different units of the summing network, we applied a perturbative approach on Eq. (1) to obtain

$$\frac{dy_i}{dt} = \alpha_0 + \frac{\alpha y_i^n}{1 + y_i^n} - y_i + x(t) + q_i(y_i)\xi_i(t) + \varepsilon \sum_{j=1, j\neq i}^{N} y_j, \tag{4}$$

where ε quantifies the degree of cross-talk. We assumed $q_i(y_i)$ not to be dependent on y_j for $j \neq i$.

Calculation of mutual information

To calculate mutual information, we solved numerically the following integral

$$\mathcal{I} = -\int_{-\infty}^{+\infty} P_{\Delta y}(s) \log_2 P_{\Delta y}(s) ds + \int_{-\infty}^{+\infty} P_{\log x}(r) \int_{-\infty}^{+\infty} P_{\Delta y|\log x}(s) \log_2 P_{\Delta y|\log x}(s) ds dr, \tag{5}$$

where we considered $\log x$ as input and Δy as output variables.

By using the Fokker-Planck equation, we calculated the probability that a unit has a given expression level, $P_i(y_i|x)$ [3]. The effective stochastic potential (ϕ_i) associated to Eq. (1)

$$\phi_i(y_i, x) = -\int_0^{y_i} \frac{f_i(s, x)}{q_i(s)^2} ds + \log q_i(y_i), \tag{6}$$

having defined f_i as the right-hand side of the corresponding dynamical equations [e.g., eqn. (1) for the bistable system] without the stochastic process. Thus, $P_i(y_i|x) = Ce^{-2\phi_i(y_i,x)}$, where C is a normalization constant so that $\int_0^{\infty} P_i(s|x) ds = 1$. In addition, when considering extrinsic noise (for the bistable system), we got

$$\phi(y_i, x) = -\int_0^{y_i} \frac{f_i(s, x + \zeta)}{q_i(s)^2} ds + \log q_i(y_i), \tag{7}$$

where ζ is a Gaussian random number with mean 0 and standard deviation q_{ex}. In case of cross-talk, we got

$$\phi_i(y_1, y_2, ..., y_N, x) = -\int_0^{y_i} \frac{f_i(s, x)}{q_i(s)^2} ds + \log q_i(y_i) - \varepsilon \sum_{j=1, j\neq i}^{N} y_j \int_0^{y_i} \frac{1}{q_i(s)^2} ds. \tag{8}$$

Finally, in the case of the excitable system, we calculated $P_i(y_i, z_i|x)$ numerically. Moreover, for the non-dynamic situation, i.e., passing signals through a threshold device described by a Heaviside function (Fig. S2), we also computed mutual information numerically.

Calculation of distance to linear response

To calculate distance to linear response (d_{lin}) [4], we averaged all realizations of y_i for a given x to obtain an average response ($\langle \Delta y_i(x) \rangle$, taking into account the initial conditions). Note that x varies uniformly between x_{min} and x_{max}. In case of the bistable system, the two output values in the deterministic regime are approximately α_0 and $(\alpha + \sqrt{\alpha^2 - 4})/2$. Then, we considered the ideal linear response as $y_{lin} = \alpha_0 + \frac{\alpha + \sqrt{\alpha^2 - 4}}{2} \frac{x - x_{min}}{x_{max} - x_{min}}$. And the calculation of the distance reads

$$d_{lin,i} = \int_{x_{min}}^{x_{max}} \left(\langle \Delta y_i(x) \rangle - y_{lin}(x) \right)^2 dx. \tag{9}$$

References

[1] Stocks NG (2000) Suprathreshold stochastic resonance in multilevel threshold systems. *Phys Rev Lett* 84: 2310-2313.

[2] Rosenfeld N, Young JW, Alon U, Swain PS, Elowitz MB (2005) Gene regulation at the single-cell level. *Science* 307: 1962-1965.

[3] Frigola D, Casanellas L, Sancho JM, Ibañes M (2012) Asymmetric stochastic switching driven by intrinsic molecular noise. *PLoS One* 7: e31407.

[4] Gammaitoni L (1995) Stochastic resonance and the dithering effect in threshold physical systems. *Phys Rev E* 52: 4691-4698.
