# Supplementary figures and images for: Genetic Redundancies Enhance Information Transfer in Noisy Regulatory Circuits
Source: PLoS Comput Biol. 2016 Oct 14;12(10):e1005156. doi: 10.1371/journal.pcbi.1005156 (PMC5065233; doi:10.1371/journal.pcbi.1005156)

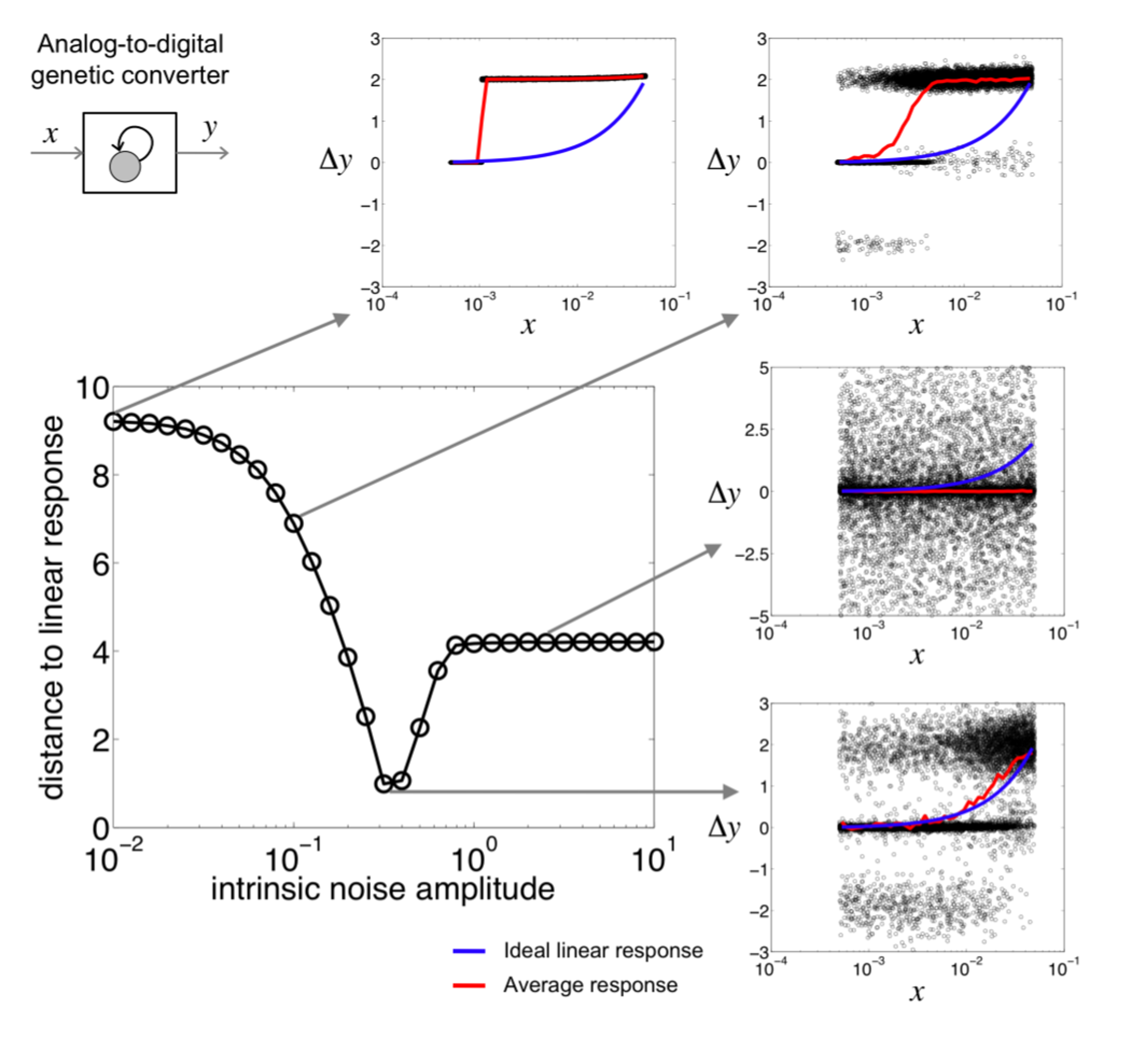

Supplement: S1 Fig — We computed the change in gene expression Δy (with respect to the initial steady state of the system) caused by the presence of the signal (x) for the bistable unit (N = 1). The distribution of the input signal values is a uniform with a mean of 0.005 and a variance that allows covering two orders of magnitude. Panels correspond to the responses of the device to 104 signal values drawn from the described distribution (black dots) and different intrinsic noise amplitudes. Average response (red curve, mean over all possible responses) and ideal linear response (blue curve) are also shown. The main plot displays the distance between the average and the linear response, which exhibits a minimum for certain amount of noise. (TIF) [file pcbi.1005156.s002.tif]

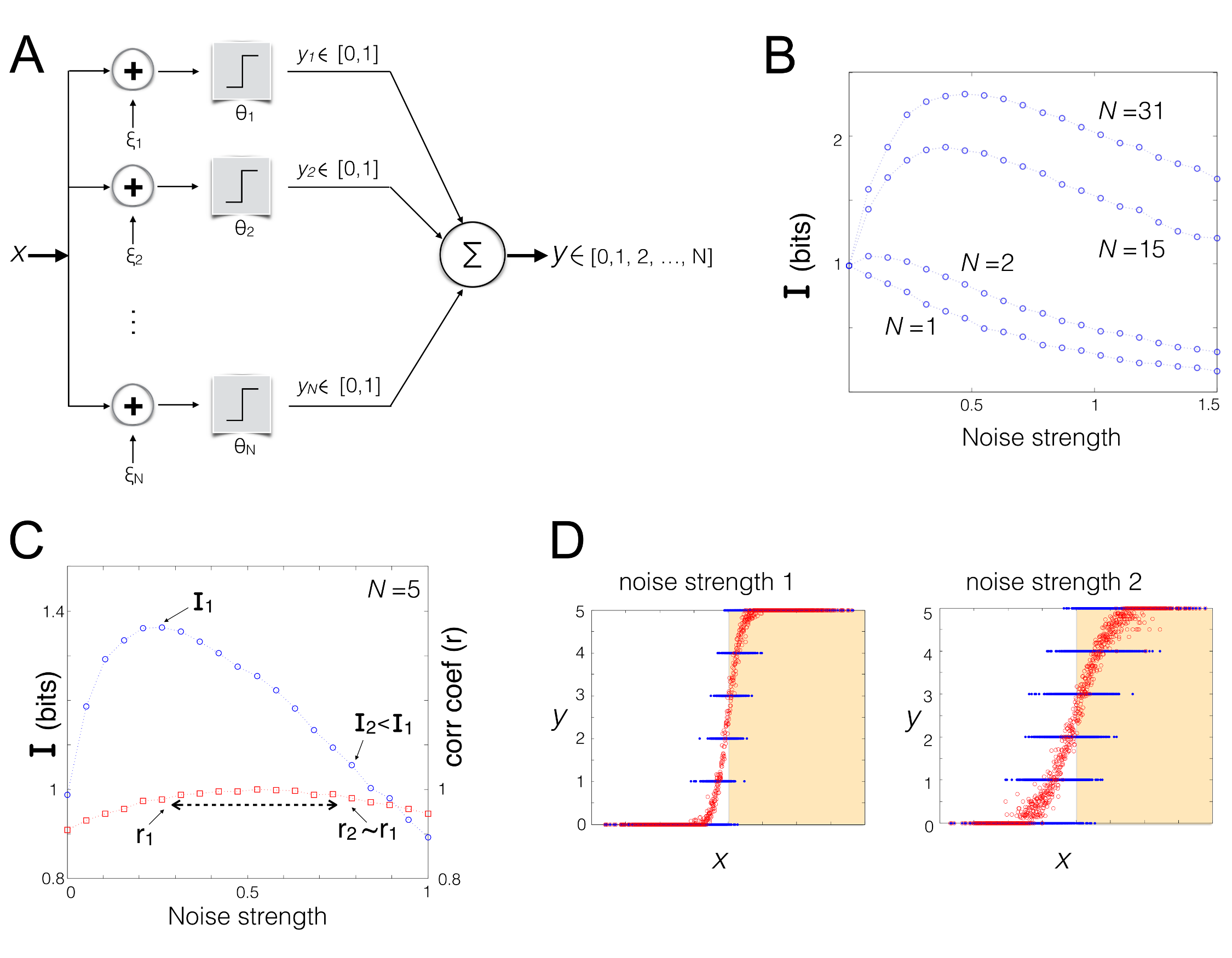

Supplement: S2 Fig — (A) A signal x acting on a summing array of threshold units i (threshold value θi), each one experiencing independent noise ξi. The output y ranges from 0 to N (the total number of units). (B) Average transmitted information through the array in (A) quantified in terms of the mutual information (I). Here, 104 signal values, drawn from a Gaussian distribution with mean 〈x〉 = 1 and σx = 1, passed through the threshold device (Heaviside function). All thresholds are set equal to the signal mean, and noise strength is determined by σi (of a Gaussian distribution with 〈ξi〉 = 0). In this situation, the signal is strongly suprathreshold, yet noise does induce a maximum (stochastic resonance) for all N > 1. (C) Quantification of information transmission using the correlation coefficient cannot distinguish between two situations with low (r1) and high (r2) noise strength as compared to the mutual information (I1 and I2, respectively). Here, we considered an array of N = 5 threshold units. (D) Response (blue) and averaged response (red) in the regimes of low and high noise in (C); values beyond the threshold highlighted in yellow. While the linear dependence between x and y is relatively similar, the information obtained about x when measuring y is not. This implies similar correlation coefficients but different mutual information. The use of I to quantify the amount of information transmission was originally discussed in [29]. (TIF) [file pcbi.1005156.s003.tif]

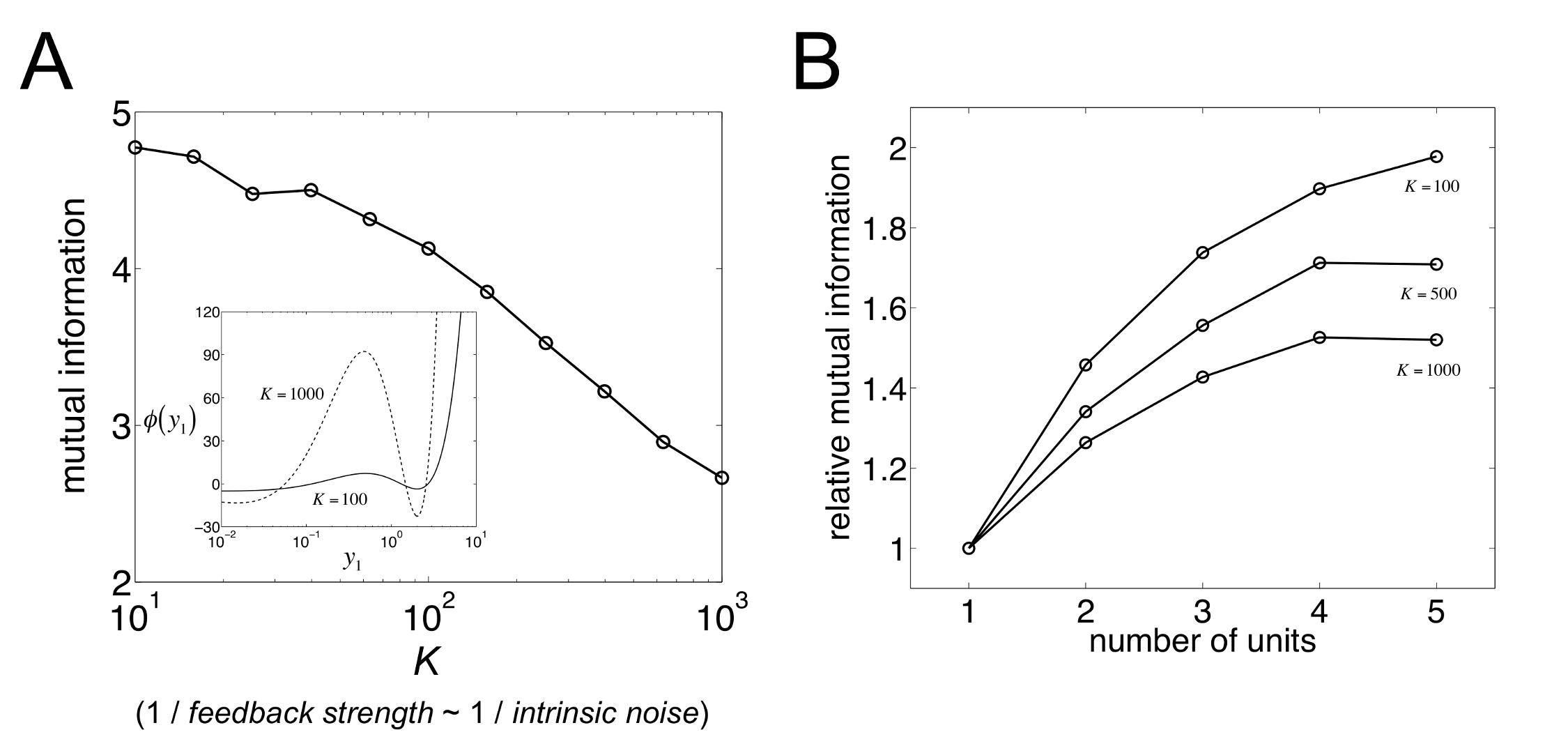

Supplement: S3 Fig — (A) Dependence of mutual information with the feedback strength (or also intrinsic noise amplitude, parameterized by 1/K) of the bistable unit. The inset shows the effective stochastic potential (ϕ) for K = 100 and K = 1000 (with N = 2). Certainly, it shows two potential wells (i.e., two stable steady states), and the threshold of the system is within (note that intrinsic noise is multiplicative, i.e., the amplitude of the stochastic fluctuations depends on the particular gene expression level). In case of higher noise (K = 100), the potential barrier is very low, indicating that it is very easy to have stochastic threshold crossings. However, in case of lower noise (K = 1000), the potential barrier is high, moderating the number of stochastic threshold crossings. The observed trend of information transfer versus intrinsic noise (higher the noise, higher the information transfer) is explained because the stochastic threshold crossings (for a continuous output variable, and to some extent) is the mechanism underlying the linearization of the response and the increase of communication fidelity. (B) Dependence of mutual information with the number of units (N) of the system. Relative mutual information is with respect to N = 1 for different intrinsic noise levels (modulated by K). This shows how the higher the intrinsic noise level, the stronger the amplification of information transfer due to genetic redundancy. (TIF) [file pcbi.1005156.s004.tif]

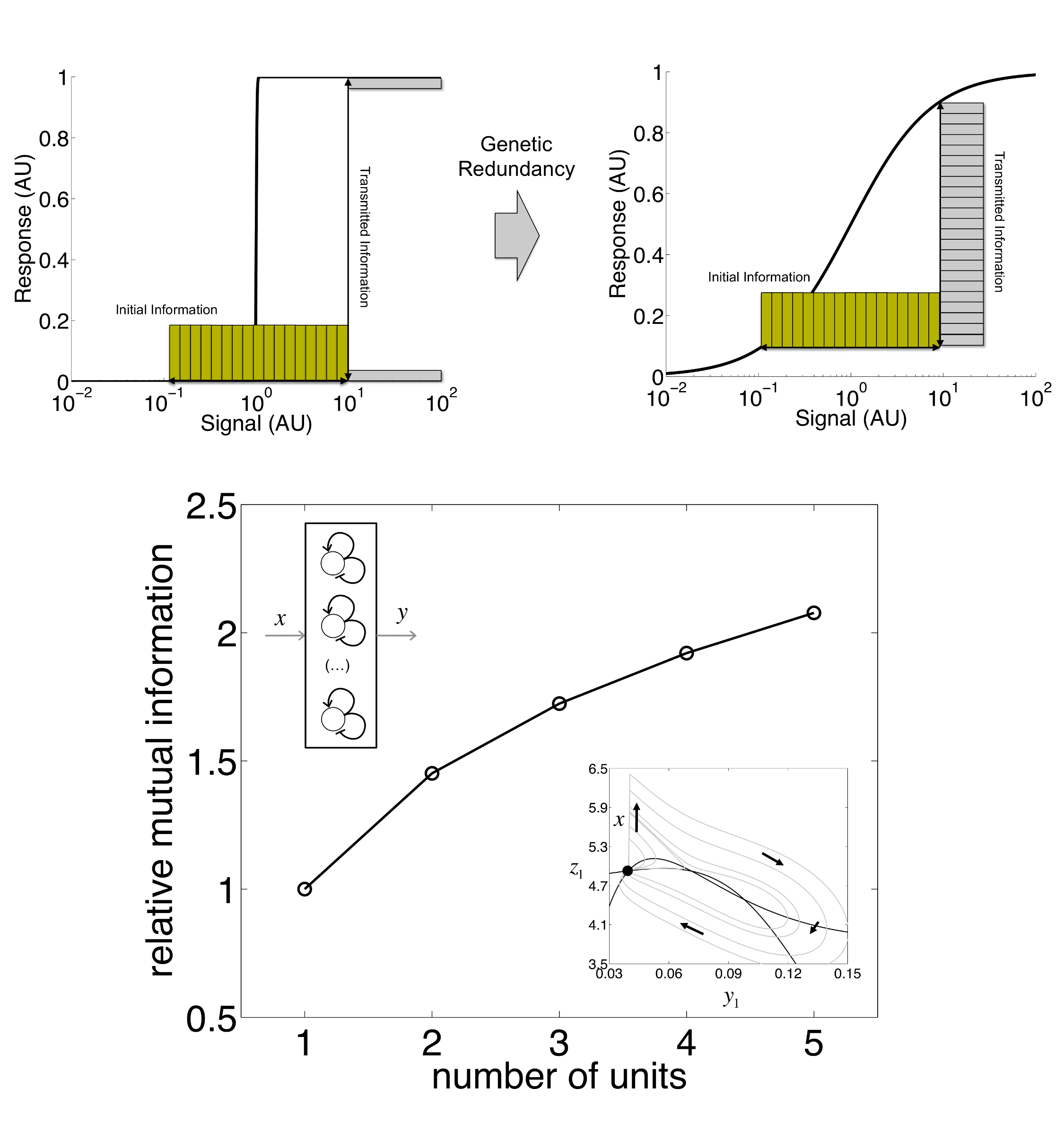

Supplement: S4 Fig — (Top) Input/output distributions depicting information transfer. The input distribution (in yellow) is assumed to be uniform (with a mean of 0.9 and a variance that allows covering two orders of magnitude). Output distributions (in gray) illustrate the processing of the signal x, either through a single copy of the threshold device (left) or an array of multiple redundant copies (right). Note that the output is Boolean, setting if the unit is excited or 0 if not. In the latter case, each unit of the array receives the same signal and the output y is the sum of all the individual responses. Redundancy effectively enlarges the alphabet of the response. This is reflected in the output distribution, and also in the linearization of the averaged stimulus-response profile (black curve). (Bottom) Relative mutual information, with respect to N = 1, for a system of N excitable units implemented with interlinked positive and negative feedbacks. According to the same input level, and in the presence of molecular noise, each unit can perform an excursion over the phase space (excitation) or not. Inset shows the phase space where the nullclines (black lines) determine the possible dynamical trajectories (gray lines, deterministic regime). Arrows indicate direction of the dynamics, and the black dot corresponds to a stable steady state. Note that a perturbation (in gene z) induces the excitation of the system provided its magnitude (x) is large enough, otherwise the system falls down to the steady state as it remains within the corresponding basin of attraction. (TIF) [file pcbi.1005156.s005.tif]

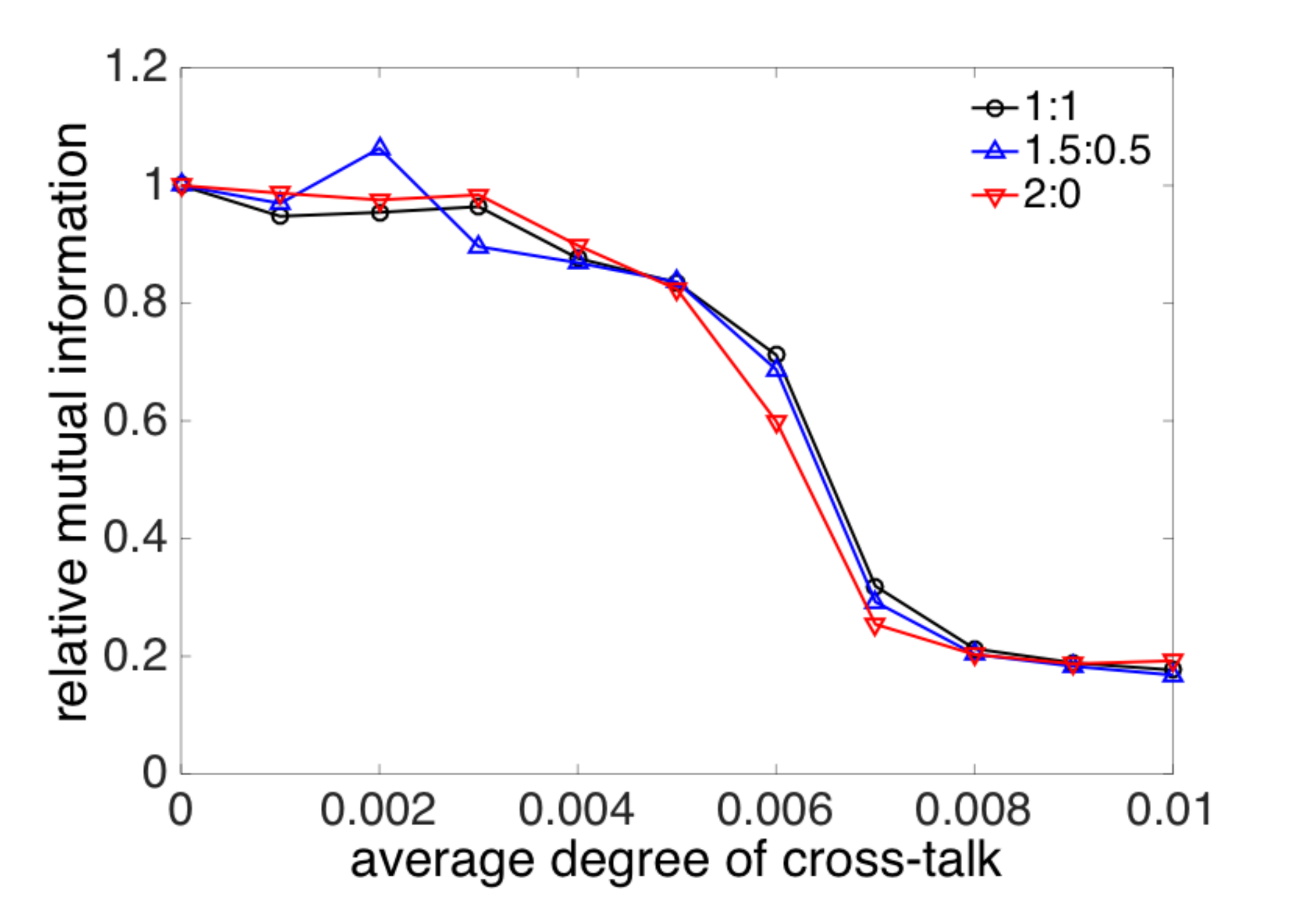

Supplement: S5 Fig — We considered a scenario of asymmetric cross-talk (parameterized by ε), i.e., gene 1 is affected by gene 2 with strength ε2 and gene 2 by gene 1 with strength ε1, with ε = (ε1 + ε2)/2. Note that the average amount of cross-talk is maintained with respect to a scenario of symmetric cross-talk. Three situations are represented (black, ε1 = ε2 = ε, symmetric; blue, ε1 = 1.5ε and ε2 = 0.5ε; and red, ε1 = 2ε and ε2 = 0), all displaying a similar trend. (TIF) [file pcbi.1005156.s006.tif]

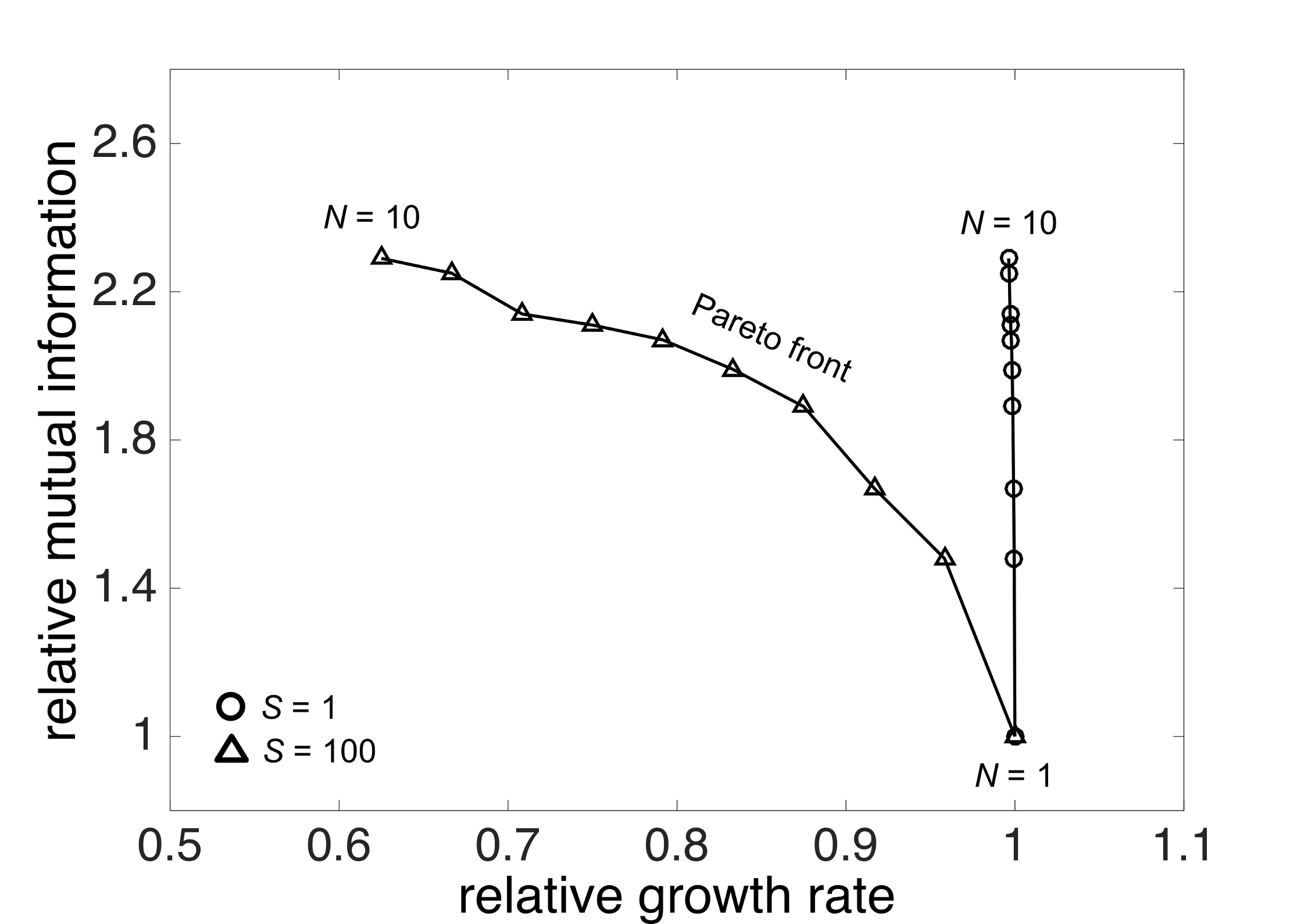

Supplement: S6 Fig — Simulations with N ranging from 1 to 10 (genetic load). For simplicity, we considered a bacterium of 5000 genes. According to previous work characterizing genetic load in bacteria [60], we can state that the relative growth rate of the cell is 1 − ϕ/0.48, where ϕ is the relative amount of unnecessary protein. In our case, ϕ = S(N − 1)/5000, where S is the number of independent systems that are implemented with redundancy. In case of a specific strategy by one system (S = 1), the genetic load is negligible. However, if redundant systems were more generally observed (e.g., S = 100), the genetic load would be considerable what leads to a Pareto front. (TIF) [file pcbi.1005156.s007.tif]
